# Co-production of a systematic review on decision coaching: a mixed methods case study within a review

**DOI:** 10.1186/s13643-024-02563-8

**Published:** 2024-06-03

**Authors:** Janet Jull, Maureen Smith, Meg Carley, Dawn Stacey, Ian D. Graham, The Cochrane Decision Coaching Review Team, Laura Boland, Sandra Dunn, Andrew A. Dwyer, Jeanette Finderup, Jürgen Kasper, Simone Kienlin, Sascha Köpke, France Légaré, Krystina Lewis, Anne Christin Rahn, Claudia Rutherford, Junqiang Zhao

**Affiliations:** 1 School of Rehabilitation Therapy, Faculty of Health Sciences, Queen’s University, Kingston, ON Canada; 2 Ottawa Hospital Research Institute, Ottawa, ON Canada; 3 Cochrane Consumer Network Executive, Ottawa, ON Canada; 4 School of Nursing, University of Ottawa, Ottawa, ON Canada; 5 School of Epidemiology, Public Health and Preventative Medicine, University of Ottawa, Ottawa, ON Canada

**Keywords:** Engagement, Evaluation, Integrated knowledge translation, Knowledge synthesis, Patient-centered research, Research co-production, Self-study, Study within a review, Systematic reviews

## Abstract

**Background:**

Co-production is a collaborative approach to prepare, plan, conduct, and apply research with those who will use or be impacted by research (knowledge users). Our team of knowledge users and researchers sought to conduct and evaluate co-production of a systematic review on decision coaching.

**Methods:**

We conducted a mixed-methods case study within a review to describe team co-production of a systematic review. We used the Collaborative Research Framework to support an integrated knowledge translation approach to guide a team through the steps in co-production of a systematic review. The team agreed to conduct self-study as a study within a review to learn from belonging to a co-production research team. A core group that includes a patient partner developed and conducted the study within a review. Data sources were surveys and documents. The study coordinator administered surveys to determine participant preferred and actual levels of engagement, experiences, and perceptions. We included frequency counts, content, and document analysis.

**Results:**

We describe co-production of a systematic review. Of 17 team members, 14 (82%) agreed to study participation and of those 12 (86%) provided data pre- and post-systematic review. Most participants identified as women (*n* = 9, 75.0%), researchers (*n* = 7, 58%), trainees (*n* = 4, 33%), and/or clinicians (*n* = 2, 17%) with two patient/caregiver partners (17%). The team self-organized study governance with an executive and Steering Committee and agreed on research co-production actions and strategies. Satisfaction for engagement in the 11 systematic review steps ranged from 75 to 92%, with one participant who did not respond to any of the questions (8%) for all. Participants reported positive experiences with team communication processes (*n* = 12, 100%), collaboration (*n* = 12, 100%), and negotiation (*n* = 10–12, 83–100%). Participants perceived the systematic review as co-produced (*n* = 12, 100%) with collaborative (*n* = 8, 67%) and engagement activities to characterize co-production (*n* = 8, 67%). Participants indicated that they would not change the co-production approach (*n* = 8, 66%). Five participants (42%) reported team logistics challenges and four (33%) were unaware of challenges.

**Conclusions:**

Our results indicate that it is feasible to use an integrated knowledge translation approach to conduct a systematic review. We demonstrate the importance of a relational approach to research co-production, and that it is essential to plan and actively support team engagement in the research lifecycle.

**Supplementary Information:**

The online version contains supplementary material available at 10.1186/s13643-024-02563-8.

## Introduction

Well-conducted and rigorous research can contribute to more effective, safe, appropriate, and sustainable health services to strengthen health systems and ultimately promote healthy societies [1, 2]; however, these potential benefits are often not realized [3–5]. In response, there has been growing support to include those who will use or be impacted by research such as patients and families, healthcare providers, health systems managers, policy makers, and other healthcare systems users (known hereafter as “knowledge users”) in research [6, 7]. Knowledge users work with researchers to co-produce knowledge and select outcomes that reflect a knowledge democracy [8, 9].

We adopt the term “co-production” in research as a collaborative approach across the research lifecycle that responds to the needs of knowledge users [6, 10, 11]. A synthesis of frameworks about engagement of knowledge users as co-producers of knowledge in health research identified 15 concepts related to the lifecycle of research studies: prepare, plan, conduct, and apply. The study authors proposed that common to co-production processes is a form of partnered negotiation that takes place at the start and throughout research studies [6]. The assumption is that there is a link between knowledge and practice, and co-production is more likely to result in the generation of knowledge that is useful and able to be used in practice and in policy. We (authors on this paper) adopt the term “engagement” in research as an arrangement in the governance of the research process where those who influence, administer, and/or use healthcare systems partner with one another as equals on a team to co-produce knowledge [12, 13]. Knowledge users and researchers work together as equals on the team to attain co-production.

There are many terms used to describe approaches to co-production including collaborative research, action research, participatory research [14], engaged scholarship [15], mode 2 research (that is, working with end users) [16], and integrated knowledge translation [14]. The term used for co-production depends on the field in which the study is being conducted, what is being produced, and who is involved [17]. Our team chose integrated knowledge translation as the research approach to structure co-production as it supports collaboration between researchers and knowledge users to develop solutions to complex issues [18]. According to the Canadian Institutes of Health Research (CIHR), integrated knowledge translation engages knowledge users as part of the research team, from defining the research question to applying the findings [16]. It is described as “meaningful engagement of the right (members of the research team) at the right time throughout the research process” [19]. Current shortcomings of co-production are conceptual (what is meant when discussing co-production) and methodological (co-production strategies and how to measure co-production) [20, 21]. In co-production, researchers and knowledge users share decisions with mutual exchange of information and learning throughout the entire research project [6], [10, 22]. Our team co-produced a systematic review to facilitate reporting on and evaluation of research processes.

### Co-production and knowledge synthesis

There is growing interest in the engagement of knowledge users in the conduct of knowledge syntheses, such as systematic reviews. Engagement of knowledge users is more likely to reduce research waste, result in evidence that addresses end-users’ needs, and improve the translation of evidence into policy and practice [23, 24]. The study of co-production processes in the conduct of a knowledge synthesis is, however, a newer field. Pollock et al. [25] assessed the conduct of systematic reviews by teams that included knowledge users across 32 studies [25]. They found that knowledge user engagement was usually reported for the initial (frame the question and plan the systematic review) and the final (interpret, publish, and disseminate findings) steps of a systematic review. It was less common to report knowledge user engagement during the conduct of the systematic review (search, screen, abstract, and analyze data) [25, 26], a finding that aligns with the general literature about engagement of knowledge users in research [6]. There is no one strategy for engagement of knowledge users with researchers in systematic reviews [27]. Devane et al. [28] identify “studies within a review” as important to address the issues related to (1) uncertainties about the evidence base for how evidence syntheses are planned, conducted, and shared, and (2) the need for high-quality evidence to inform decisions for the conduct of evidence syntheses [28]. Little is known about team members’ experiences of using a co-production approach to conduct a systematic review.

### The research context

We are a team with members from eight different countries and include those who experience chronic health conditions (patient/caregiver partners, here referred to as Cochrane consumers) and researchers, many of whom hold additional roles (for example, healthcare professionals, health systems administrator, educator, role with a health advocacy organization). We hold common interests in interventions to support shared decision-making processes for people seeking healthcare for themselves or for family members. Shared decision-making is a process where the patient and their clinician(s) work together to use the best available evidence, clinical expertise, and the patient’s informed preferences to make a decision that is tailored to the individual patient needs [29, 30]. An intervention called decision coaching holds potential to facilitate shared decision-making processes [31]. We decided that a systematic review was needed to understand the effectiveness of decision coaching and to identify research gaps. In addition, we wanted to understand how to conduct the systematic review to generate evidence in ways that can be considered useful and able to be used in policy and practice and set up a study within a review. Thus, we sought to conduct and evaluate the co-production of a systematic review on decision coaching for people facing treatment and screening healthcare decisions for themselves or family members.

Our study objectives were to:

1. describe the co-production approach to conduct the systematic review;
2. explore the experiences of team members with the co-production of the systematic review;
3. elicit perceptions of team members about the co-production of the systematic review.

## Methods

### Study design

We chose to evaluate ourselves to learn from our own systematic review team members’ experiences and perceptions, that is, to conduct a self-study of belonging to a co-production research team [32, 33]. We consider our study to be an example of a study within a review [28] as there is uncertainty in the methodology for engaging knowledge users in co-production, as well as the methods for evaluation of co-production. Here, we describe an approach to the co-production of a systematic review and present an evaluation of methods we used for organizing the co-production of a systematic review. We bound our work in time and context, and report on events that occurred between August 2019 and February 2022 when we conducted a mixed methods case study in the context of an international, interdisciplinary team conducting co-production of a systematic review [34–36]. We structured our work with the pragmatic intent to produce knowledge that is useful and could be used in practice and policy by team members and wider networks interested in shared decision-making interventions. The University of Ottawa’s Research Ethics Board approved the self-study (#20,190,408-01H).

**Fig. 1:**
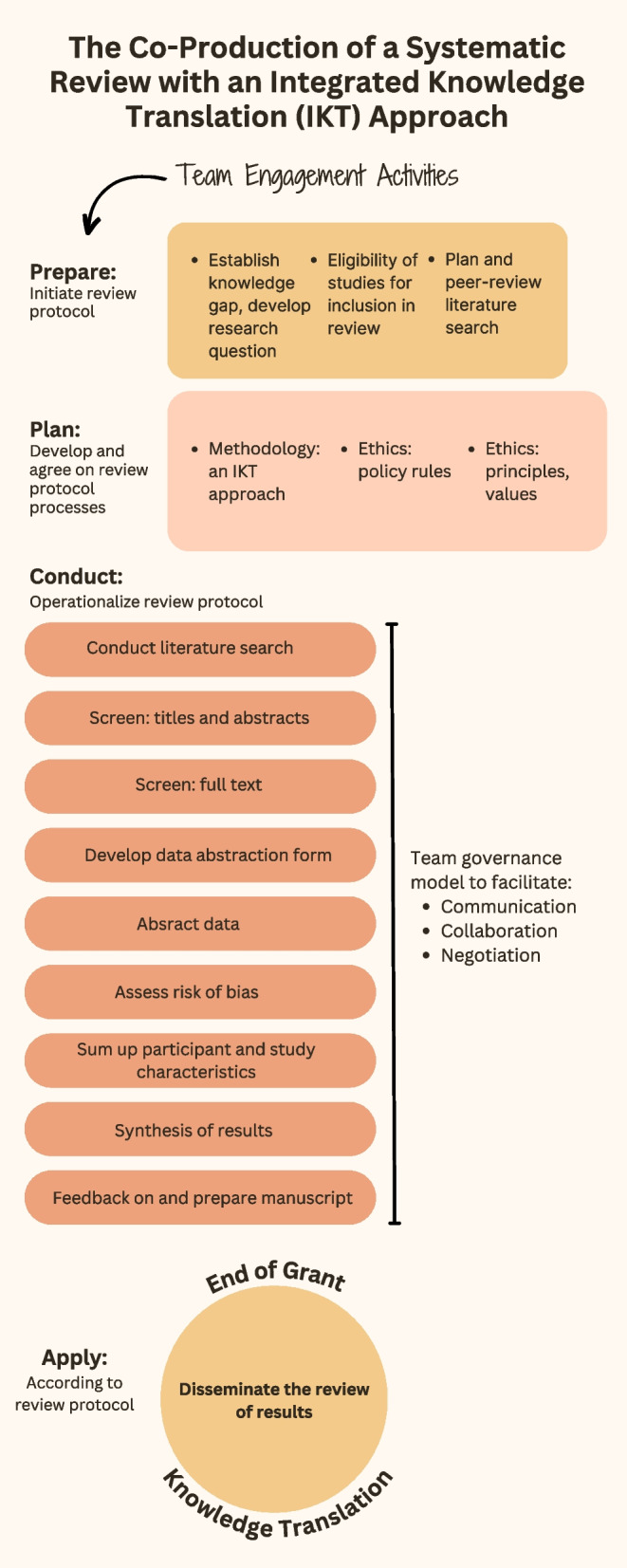
The co-production of a systematic review with an integrated knowledge translation approach

We used the Collaborative Research Framework [37] to support an integrated knowledge translation approach, to guide partnering of knowledge users and researchers to prepare, plan, and conduct the systematic review. We were limited to dissemination of the systematic review findings due to time and complexity of the task, and did not complete the “apply” step [6] (Fig. 1).

The framework assumption is that facilitation of integrated knowledge translation processes is possible with the preparation of team members to engage in iterative processes of knowledge exchange and learning throughout the research study and following replicable research steps: first, establish the guiding features for co-production with a team governance structure; and second, define research actions to be operationalized by the team to support the co-production of research evidence [16]. The framework has been used previously to guide research processes that include knowledge synthesis [38–42].

To ensure transparency and completeness in our work, we used the Guidance for Reporting Involvement of Patients and the Public 2 (GRIPP2) to report on the systematic review team engagement as it is a checklist to report on engagement in general health and social care research [43] (Supplemental file #1). We report on our self-study with the Standards for Reporting Qualitative Research (SRQR) reporting guideline [44] (Supplementary file #2).

### Setting, participants

Prior to the conduct of the systematic review, two authors on this paper (JJ, DS) initiated processes to “prepare” and “plan” the systematic review to better understand decision coaching as an intervention [31]. JJ and DS held conversations with Cochrane consumers and researchers, many who held dual roles as healthcare providers, educators, policy makers, and all with common interests on the topic of decision coaching. For example, a meeting was held with Cochrane consumers who were familiar with systematic reviews as peer reviewers, to explain the project, opportunities for involvement, and their preferences. JJ and DS then assembled a team to prepare and plan the systematic review proposal (“the team”). With one person coordinating activities (JJ), the team engaged in an iterative series of conversations to negotiate:

1. common language, definitions, and concepts to describe our systematic review topic (that is, decision coaching);
2. the systematic review question, approach, and procedures;
3. the strategies for working together, that is, to co-produce the systematic review.

The team described the systematic review, and plans for self-study to evaluate co-production processes, in a funding application. While we considered all team members to be of equal value, we did not expect that they all contribute in the same manner. Team members brought a range of skills that could be helpful for activities in relation to their knowledge, occupational roles, and in relation to life experiences. Throughout the review, training was offered to team members on an “as-needed” basis and often took the form of one-on-one conversations to explain concepts or review steps.

After our team received funding from the Canadian Institutes of Health Research (CIHR) (April 1, 2019), the “conduct” phase of the systematic review began. We hired a study coordinator (MC) to support the systematic review and study within a review tasks. Compensation to support the participation of Cochrane consumers was arranged according to CIHR guidelines [45]. The team disseminated systematic review findings following a plan developed for the systematic review protocol, with presentations [46, 47], and following publication of the systematic review (November 2021) [48] with plain language reports [49–51], and communications to network partners. The systematic review and study within a review tasks were coordinated through the University of Ottawa and Ottawa Hospital Research Institute, in Ottawa, Ontario, Canada.

We (full team) agreed to the conduct of a study within a review to learn from team members’ belonging to a co-production research team. Systematic review team members with expertise in the use of an integrated knowledge translation approach to co-production that includes a Cochrane consumer (MS, IDG, DS, JJ) formed a core group that included the study coordinator to collaborate in the development, conduct, and analysis of the study. The full team was consulted by the core group on the final plans for the self-study [52]. All 17 team members (excluding the study coordinator) of the systematic review were eligible to participate in the study. The study occurred immediately before initiation of and then after the “conduct” phase of the systematic review.

### Procedures for self-study recruitment, engagement and data collection

The study data sources included surveys and documents related to the systematic review. Prior to the conduct of the systematic review, and to begin the study, the study coordinator sent an email to all team members inviting them to participate. Of those who indicated interest in participation, the study coordinator sent further information explaining the study, data management (protection and storage), and seeking their consent to participate. Team members were informed that agreement or refusal to participate would have no effect on their status as a member of the team.

The team members who provided consent were asked to participate in a survey. In the survey, they were asked to respond to open- and closed-ended questions on preferred level of engagement during the conduct of the systematic review, and their experiences and perceptions co-producing the systematic review. Participants in the study were sent an email with a link to a baseline survey. At 1 and 2 weeks after the initial request, email reminders to complete the baseline survey were sent to participants [53]. Then, the study coordinator invited participants to be involved in the various steps of the systematic review based on their preferred level of engagement reported in the baseline self-study survey; the remainder of the systematic review team members were sent a general invitation to be involved at the various steps of the systematic review. The study coordinator arranged meetings and engagement in the tasks. If participants wanted to co-lead a step of the systematic review, they were invited to join the executive committee to conduct tasks. If they wanted to participate in a particular step of the systematic review, they were provided with the resources to participate (for example, access to Covidence to screen studies). Participants were reassured that there would be no repercussions if their availability or interest in participation with the steps of the systematic review changed from the baseline survey to when the systematic review task needed to be done. Upon completion of the systematic review, an end of self-study survey was administered to participants with the same procedures. We report on our survey [54] with the Checklist for Reporting Results of Internet E-Surveys (CHERRIES) [55] (Supplementary file #3). Members of the core group that led the conduct of the study within a review (MC, JJ) collected systematic review documents (for example, meeting notes, systematic review paperwork, researcher journal).

### Survey instrument

The core group in the development and conduct of the study with the study coordinator led the development and pilot testing of the online surveys. We could not identify an instrument to evaluate how well our team worked on conducting the systematic review. Overall, the instrument consisted of seven pages of questions, with between one to 17 items per page of the survey, for a total of 39 items. The survey took between 15 and 30 min to complete and participants had the option to answer or skip questions as they preferred.

We view the process of co-production to require consideration of the people in the research partnership, and the contexts in which they are situated, and wanted our evaluation to reflect their realities as participants:

1. Our approach to co-production involved assessment of participants’ preferred levels of engagement (that is, role) in the systematic review steps (baseline self-study survey) (for example, reviewing the search strategy, screening citations), and confirmation of participants’ actual levels of engagement (end of self-study survey), based on Arnstein’s Ladder of Citizen Participation and the Strategy for Patient-Oriented Research – Patient Engagement Framework [56, 57] and previous research [58, 59] about engagement in research. There were options for preferred level of engagement for each systematic review step, and open-ended questions that asked for any additional comments about their level of engagement in the steps of the systematic review. At the completion of the systematic review, participants were asked questions about their satisfaction with each step of the systematic review (end of self-study survey) [40].
2. To explore participants’ experiences with the co-production of the systematic review, we used the 36 item Partnership Indicators Questionnaire (PIQ) instrument adapted with permission of the PIQ lead author [60–62]. The PIQ is designed to assess the performance of researcher-health policy maker partnerships. Three lists of indicators include the following: common partnership indicators, early partnership indicators, and mature partnership indicators. The dimensions include the following: (common partnership indicators) communication, collaboration in research, dissemination of research, (early partnership indicators) research findings, negotiation, partnership enhancement, (mature partnership indicators) meeting information needs, level of rapport and commitment. The selected PIQ questions focused on the concepts of communication, collaboration, and negotiation and included an open text option for additional comments (baseline, end of self-study surveys).
3. We elicited the perceptions of participants about co-production of the systematic review with closed and open-ended questions with a focus on challenges, benefits, and impacts, at baseline and upon completion of the systematic review (end of self-study survey) [40, 63].
4. We collected participant demographic characteristics.

### Study documents

We utilized team documents that included meeting notes, systematic review paperwork, and a researcher journal, to conduct the document analysis method [64]. The aim was to report on engagement of the team members, following completion of the systematic review. We used the **A**uthors and** C**onsumers **T**ogether** I**mpacting on eVidencE (ACTIVE) framework to describe and report on Cochrane consumer engagement across team activities in the steps of systematic reviews. The ACTIVE framework is used to define whether and how knowledge users are engaged in systematic reviews, for example, the method of recruitment, the approach, format, and stage of engagement [25, 26].

### Analysis

The core group in the development and conduct of the study with the study coordinator conducted the analysis. The study coordinator provided frequency counts for quantitative survey data. One researcher (JJ) conducted the frequency counts of open-text responses, and content analysis. A panel of second reviewers (MS, MC, DS, IDG) confirmed the work through interpretation and discussion [65] with reflection on concepts related to engagement [6]. Survey responses to closed-ended questions were tabulated; content analysis was used to analyze responses to open-ended questions, which involved segmenting responses by topics and into categories. Each question was considered to be a topic and the responses and development of codes defined the content in each category [66]. We report the frequency counts of the type of responses with illustrative quotes.

The team documents were reviewed for evidence that the systematic review was co-produced. One researcher (JJ) with the support of a second (MS) engaged in a three-stage analysis process of skimming, reading, and interpretation [66] to reach consensus, reported using the ACTIVE framework [25], and confirmed by a panel of reviewers (MC, DS, IDG). The analysis includes the ways in which decisions were made, meetings conducted, and information communicated [25]. While data analyses were initially conducted separately, findings were interpreted by corroborating quantitative and qualitative findings [67]. As part of the mixed methods, quantitative data findings were identified as the main data source with qualitative findings used to supplement and explain the quantitative findings.

## Results

Data collection commenced after we received ethics approval in August 2019 to evaluate the co-production of the systematic review and was completed in February 2022, after publication of the systematic review by the Cochrane Library [31]. First, we provide the characteristics of participants. Then, we describe the co-production approach to the conduct of the systematic review (the context for co-producing the systematic review, and participant engagement) (objective 1), explore the experiences of team members with co-production of the systematic review (objective 2), and elicit perceptions of team members about co-production of the systematic review (objective 3).

### Characteristics of the participants

Of 17 team members, 14 (82% response rate) consented to participate and completed the baseline self-study survey, and of the participants 12 (86% response rate) completed the post self-study survey. We report on the 12 participants who completed both surveys.

Of 12 participants, most self-identified as women (*n* = 9, 75%), were born between 1950 and 1980, and all had graduate level of education (see Table 1). There were seven researchers, four researcher trainees, two clinicians, two patient/caregiver partners (Cochrane consumers), and three researchers who reported that they held additional roles (administrator in a health system, health educator, patient advocate with an organization) and team members may have belonged to more than one category. Participants reported a broad range of experiences with decision coaching, systematic review methods, and shared decision-making. All participants indicated that they would be able to act on the findings of the systematic review, and two indicated that they might also be impacted by the findings.

**Table 1:** Characteristics of participants (*n* = 12)

Characteristic		*n*	%
Decade of birth	1950s	2	17
	1960s	3	25.0
	1970s	3	25.0
	1980s	4	33
Gender identity	Woman	9	75.0
	Man	3	25.0
	Other	0	0
	Prefer not to say	0	0
Current level of education	Graduate	12	100
Role(s) on the research team (may belong to more than one category)	Researcher	7	
	Trainee (e.g., graduate student, post-doctoral fellow)	4	
	Clinician	2	
	Patient/caregiver partner	2	
	Other (i.e., health system administrator, educator, health advocacy organization)	3	
Experience with decision coaching	No previous experience	0	0
	Beginning to learn about decision coaching	2	17
	Received decision coaching as an intervention in a health system	0	0
	Deliver decision coaching to someone making a decision	9	75
	Participate in training	4	33
	Develop training programs about decision coaching	7	58
	Provide(d) training programs about decision coaching	6	50
	Participate(d) in research about decision coaching	2	14
	Conduct research about decision coaching	8	67
	Develop or promote policy for decision coaching	2	17
	Other (develop interventions, coauthor paper)	2	17
Experience with the steps involved in conducting a SR	No previous experience	0	0
	Read or reviewed abstracts/consumer summaries of a systematic review(s)	12	100
	Conducted searches in databases and removed duplicates	9	75
	Screened titles and abstracts of citations	11	92
	Pulled full text articles	10	83
	Screened full text of citations	10	83
	Searched grey literature sources	9	75
	Abstracted data into data collection forms	10	83
	Assessed risk of bias of included studies	10	83
	Conducted analysis	9	75
	Drafted the SR article(s)	10	83
	Provided feedback on the article(s)	10	83
	Shared SR findings with those who can use them (disseminate)	9	75
	Peer-reviewed a SR article(s) for a journal	10	83
Knowledge of tools and approaches related to the shared decision-making process	Shared decision-making (process)	11	92
	Patient decision aids (tools)	11	92
	Decision coaching (approach)	10	83
	Other** (**e.g., used decision aids, SDM training, interventions to support SDM for patients)	3	25
Which statement best reflects your role as it pertains to the results of the systematic review on decision coaching			
I am able to act on the findings of the review		12	100
I may be impacted by the findings of the review		2	17
I am neither able to act on the findings nor will I be impacted by the findings of the review		0	0
None of the above		0	0
Prefer not to say		0	0

Objective 1: describe the co-production approach to the conduct of the systematic review

### The context for co-producing the decision coaching systematic review

We conducted all study tasks remotely, due to the pandemic. To operationalize co-production of the systematic review, we (the team) assembled and self-organized a governance structure with an executive team (MS, SK, DS, JJ), of which one was a Cochrane consumer, and a Steering Committee consisting of the remaining 13 team members. The study coordinator supported the team. The executive team was tasked with operationalizing the systematic review and the Steering Committee with providing direction to the systematic review and engagement in systematic review tasks as they preferred. In the 15 months of the systematic review, the executive team met online every 2 weeks and the Steering Committee met online four times. The team members committed to roles and processes for systematic review governance and negotiated terms of reference document based on a previously developed format [40] (Supplementary file # 4).

The team agreed on features for the conduct of the systematic review in two stages: (1) ethical guidance, theoretical perspective, and (2) research actions. The team agreed on concept definitions for shared decision-making and decision coaching [16, 31, 68]. Finally, the team agreed upon an integrated knowledge translation approach to structure research actions and engage team members in co-production with support strategies such as communication, collaboration, and negotiation [61]:

1. During the conduct of the systematic review, the study coordinator and executive team maintained communication with the Steering Committee through email to support engagement in systematic review team activities at the level participants had indicated they wanted to be involved (individual 1:1, and group). In addition, the study coordinator shared systematic review progress summaries monthly with the team.
2. The study coordinator used the information from the baseline self-study survey to provide a structured approach to collaboration, with an individualized approach to engaging team members for the systematic review steps.
3. The team members had the opportunity to indicate their preferred level of engagement in the systematic review throughout the study.

We adjusted our plans for assessment of preferred and actual levels of engagement with each step in the systematic review. We originally asked participants about their preferred levels of engagement for all eleven systematic review research steps. There were four steps in which we did not end up involving team members who volunteered for those steps: (1) “conduct literature search” was done by the librarian and did not engage team members; (3) “pull full text articles” was done by the study coordinator; (8) “conduct analysis” consisted of the study coordinator adding extracted outcome data to Review Manager [69] (RevMan Web 2023) and leading the conduct of meta-analyses according to the protocol with support of other team members (DS, JJ), and followed with team review and confirmation; and (9) “draft the systematic review article” was co-led by two team members (JJ, DS). For the study of the systematic review conduct, as we were only just initiating the systematic review and the baseline results (although all extremely positive) were not a relevant indicator of team function, we only report the end of systematic review survey results for satisfaction with the level of engagement.

### Participant engagement

Participants’ actual roles in the systematic review steps were mostly consistent with their preferred roles but there was a slight overall move towards greater engagement (“invite me [to participate]”) (see Table 2).

**Table 2:**
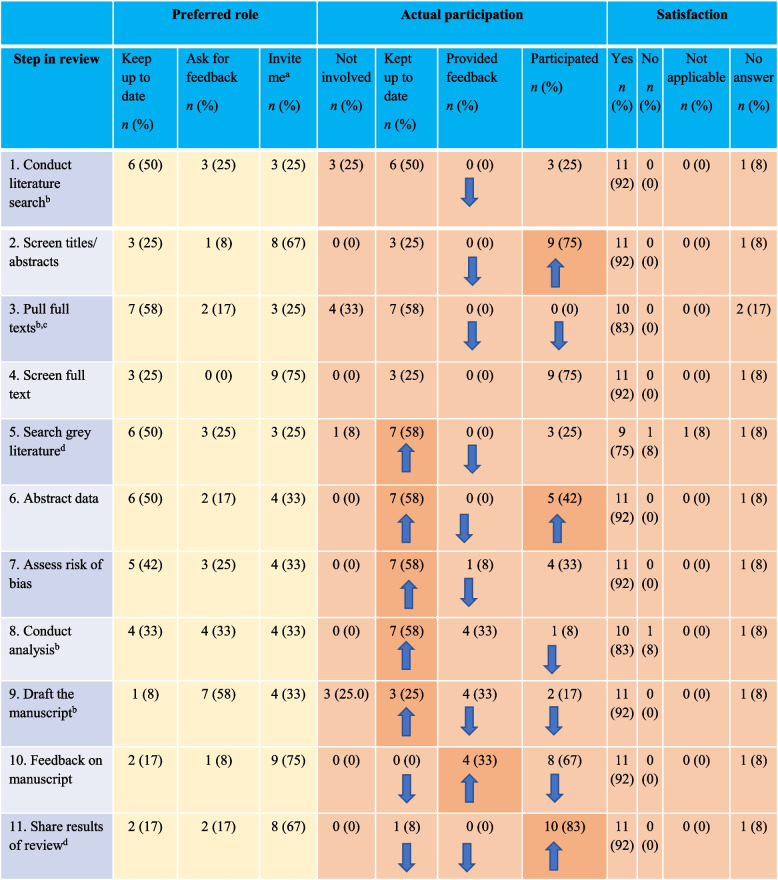
Participant preferred, actual role and satisfaction in the conduct of the systematic review (*N*=12)

Arrows signal increase or decrease in participation, actual participation reported by participant and confirmed by study coordinator

^a^For “invite me” we merged “participate” and “co-lead”

^b^Team members were not invited to participate in this step

^c^For actual participation, one team member did not respond

^d^For actual participation, one person indicated I do not know/unsure

Eight participants provided more detailed responses to the open-ended question that asked for any additional comments about their level of engagement in the steps of the systematic review. Four participants explained their decision to engage with the systematic review tasks due to a positive experience with the team (for example, “*opportunity to reach out if we had any questions*,” a “*collegial approach*” and “*wonderful experience in knowledge synthesis and IKT*,” “*many learning opportunities and the team made it clear that it was a mutual learning experience which was very gratifying*”). Of these eight participants, three reported that their level of engagement was due to the project management of the systematic review: “*great project management*,” “*project team leads’ efforts to engage team members…for example, email updates* [newsletters] *were effective for the progress of the review*,” “*I participated in regular team calls*.” Two participants identified factors which impacted their engagement: one participant identified perceptions of their role with the systematic review process, “*I was actually able to participate more than I had envisioned*”; the other alluded to factors external to the systematic review that limited engagement, “I *would have liked to have had the time and opportunity to prioritize having been even more involved in the project*.”

Of 12 participants, 11 were satisfied with their level of engagement at each step of the systematic review and one did not respond. There were two exceptions: for the step “search grey literature” with slightly lower participant satisfaction scores with most satisfied (*n* = 9, 82%), one indicating they were not satisfied (9%) and one indicating “not applicable” (9%), and “conduct analysis” with most satisfied (*n* = 10, 90.0%) and one indicating they were not satisfied (9%). If the systematic review was to be conducted again, eight indicated wanting to have the same level of engagement and four indicated that they would want to be more engaged. Five participants indicated that they were very satisfied and seven totally satisfied, with the extent to which they were engaged in the project.

Using the ACTIVE framework to report on the systematic review team engagement, team members were recruited by the study coordinator to engage in the conduct of the systematic review using a closed (by invitation) strategy. The mode of engagement was defined as continuous, with team members engaged during all steps of the systematic review, and with leading or controlling levels of influence on the conduct of the systematic review [25] (Supplemental file #5).

Objective 2: explore the experiences of team members with co-production of the systematic review

Participants reported on team processes that supported their ability to participate in co-producing the systematic review, these processes being communication, collaboration, and negotiation. With few exceptions, self-study participants reported positive experiences, mostly strongly agreeing or agreeing with the survey statement [61]. None responded with disagree or strongly disagree (see Table 3).

**Table 3:** The participants’ perceptions of the co-production approach to conducting the systematic review – results from the partnerships indicator questionnaire (*n* = 12)

	Strongly agree	Agree	Neutral*
**Communication**			
On-going	12	0	0
Communication involved email, web-based telecommunications (e.g., Zoom), and/or face-to-face meetings	11	1	0
Same people over the life of the project	12	0	0
A common language used	9	3	0
Explicit roles, expectations, deliverables	9	3	0
Valued each other’s contributions	11	1	0
Acknowledged in project documents	12	0	0
Open text: “Communication was consistent – concise and informative” (1/12, 33%)			
**Collaboration**			
Team members contributed to drafting the report for Cochrane	7	5	0
Team members participated in the SR to ensure relevance of the research	12	0	0
Feedback about report was provided before submitted to Cochrane	12	0	0
Response to feedback prompt	10	2	0
Report submitted to Cochrane was acceptable to all (1 not applicable)	10	1	0
Open text: “Research team provided accommodations to a team member to ensure their meaningful contributions” (1/12, 33%)			
**Negotiation**			
Roles and responsibilities of team members were documented	8	3	1
Written terms of reference for the research project	7	3	2
Requirements for steps in the project, deliverables, and time lines documented	9	3	0
Team members made their needs explicit	9	3	0
Team members document the above (negotiation) needs	8	3	1
Open text: “In-person meeting with co-PI to negotiate my role was very helpful and set the tone for the review” (1/12, 33%)			

^*^No participant indicated a response of disagree or strongly disagree

One participant provided a comment in the open text about communication: *“communication was consistent – concise and informative.”* Another participant related a comment about team collaboration: *“Research team provided accommodations to a team member to ensure their meaningful contributions.”* While most participants strongly agreed or agreed they had positive experiences co-producing the systematic review, some participants indicated a neutral response for three items about documentation of roles and responsibilities for their participation in the study processes, systematic review terms of reference, and documentation of team member needs. In addition, one participant reported that the *“In-person meeting with co-PI to negotiate my role was very helpful and set the tone for the review*.*”*

Objective 3: elicit perceptions of team members about co-production of the systematic review

Overall, participants indicated that the integrated knowledge translation approach was successful in leading to co-production of the systematic review. Participants reported a strong positive team experience (see Table 4).

**Table 4:** Results of six open-ended team survey questions about experiences with an integrated knowledge translation approach to conducting a systematic review (*n* = 12)

Question	Response
1. To what extent was the systematic review process truly one of co-production?	Large extent (2/12, 16.7%) Very large extent (10/12, 83.3%)
2. How do you know the systematic review process was truly one of co-production (or not)?	Tools to structure collaboration (2/12, 17%): “our guiding framework, terms of reference…” Collaborative activities (8/12, 67%): “engagement of members during zoom call[s]” Engagement of those in different roles (8/12, 67%): “we had a patient on the executive. We had team members that had various roles” Knowledge from the review shared (1/12, 8%): “we have taken relationships with stakeholders very seriously, and worked to convey results in meaningful ways”
3. Would you have changed anything about how a co-production approach was used in the (systematic review) study? Please explain	1. No (8/12, 67%): a. “Followed model for co-production” (2/8, 25%) b. “Effective/efficient” (2/8, 25%) c. “People chose their preferred participation” (1/8, 13%) d. No comment (3/8, 37.5%) 2. Not sure (4/12, 33%): a. “More conversation with team about how to work together” (1/4, 25%) b. “Consider inclusion of consumer panel” (1/4, 25%) c. No comment (2/8, 25%)
4. What were the challenges of working in a way that supports co-production?	1. The logistics of including a range of people in the team (5/11, 45%): “We are many people and from the whole of the world.” 2. Manage team needs for participation (3/11, 27%): “…ensure everyone was able to participate at the level they wanted to be involved.” 3. No challenges (4/11, 36%): “I experienced no challenges” 4. No response (1/12, 8%)
5. What do you perceive as the benefits or impacts of using a co-production approach during the conduct of the systematic review?	Inclusion of different perspectives (8/12, 67%): “inclusion of diverse perspectives” Relevance and use (4/12, 33%): “optimize the relevance, the usefulness…of the systematic review [systematic review] output” Increase research impact (3/12, 25%): “more diverse dissemination to various audiences” Impact on structure for engagement with review (2/12, 17%): “major decisions during protocol…small judgements and decisions during the conduct of the review”
6. If you had the opportunity to work on another project with the same team, do you think you would? Why or why not?	1. Yes (11/12, 92%): a. Clear roles (2/12; 17%): “appreciated the flexibility to be able to contribute based on my interests and strengths” b. Trust (3/12, 25%): “felt welcomed and valued” c. Opportunities to participate (2/12, 17%): “opportunities to comment and participate along the way” d. Objectives achieved (3/12, 25%): “…resulted in a high quality product” e. Good experience (6/12, 50%): “very positive experience” 2. No (0/12, 0%) 3. Prefer not to say (1/12; 8%): “I’m choosing this one as it’s a ‘maybe’. We did the best we could given the circumstances, next time we should include more consumers/community members”
Do you have any additional comments?	“Good experience” (5/7, 71%) “Appreciate opportunity to be on team” (1/7, 14%) “Appreciate opportunity to comment on experience” (1/7, 14%) No response (5/12, 42%)

Eight participants reported that collaborative activities and engagement of team members align with a co-production approach: *“We had a patient on the executive. We had team members that had various roles*.*”* Eight participants reported that they would not change anything about the co-production approach, and of the four that indicated being unsure, two suggested more opportunities for communication and inclusion.

Challenges to co-production were reported by five participants to be due to team logistics: “*We are many people and from the whole of the world*.” Four participants reported not being aware of any team challenges. Eight participants described the inclusion of a range of perspectives as the benefit of the co-production approach, and four participants reported the co-production approach was a way to *“optimize the relevance, the usefulness of the systematic review output*.*”* Eleven of the 12 participants indicated that they would work on another project with the same team. When asked for additional comments five participants reported a strong positive or “good experience.”

## Discussion

Our international and interdisciplinary team that included two Cochrane consumers organized study governance and agreed on research actions and strategies to support co-production of the systematic review about decision coaching. Overall, our results indicate that it is feasible to use an integrated knowledge translation approach to conduct a systematic review. Participants reported satisfaction with their engagement in the steps of the systematic review and positive experiences with and perceptions of team processes. Our study reveals that planning and actively supporting team member engagement in the research lifecycle led all team members to perceive the systematic review as co-produced.

### A relational approach to research is crucial for the success of co-production

Our study demonstrates the importance of a relational approach for co-production, meaning that there must be interactive processes among team members that leads to relationship building. Our findings about the relational nature of co-production align with recent work on integrated knowledge translation guiding principles that include the following [19]: (1) partners (knowledge users with researchers) develop and maintain relationships based on trust, respect, dignity, and transparency; (2) partners share in decision-making; (3) partners foster open, honest, and responsive communication; (4) partners recognize, value, and share their diverse expertise and knowledge; (5) partners are flexible and receptive in tailoring the research approach to match the aims and context of the project; (6) partners can meaningfully benefit by participating in the partnership; (7) partners address ethical considerations; and (8) partners respect the practical considerations and constraints of all partners.

In our study, team members who indicated wanting to be involved with systematic review steps at the start of the study reported high satisfaction with their levels of engagement in the systematic review and attributed their positive experiences with the team for their levels of engagement. Some participants indicated not being satisfied with the steps for which there was limited opportunity for engagement. The engagement of knowledge users with researchers in research has distinct and important benefits that include the generation of knowledge that is useful and able to be used in practice and policy [23, 24, 70, 71]. Based on our findings, we propose that there is potential for very positive and enjoyable inter- and intrapersonal benefits for those who participate in co-production.

### Team engagement in the research lifecycle of a systematic review

It is essential to plan for and actively support collaboration, communication, and opportunities to negotiate engagement of team members who want to co-produce a systematic review. We chose an integrated knowledge translation approach to create opportunities for team members to share decisions, exchange information, and learn from one another. Other studies have described teams as challenged by co-production, due to the potential for a lack of clarity about study motivations and outcomes [72]. There is also the potential for those in the research partnership to experience risks that are practical (for example, time, money), personal (for example, interpersonal conflict, stress), and professional (for example, impacts on reputation) [21, 73, 74]. The participation by team members in the conduct of the systematic review was voluntary, and our team benefitted from an experienced study coordinator. When work or personal factors had an impact on team members’ ability to participate, the study coordinator was able to manage the logistics of scheduling meetings or study tasks to accommodate team members’ changing availabilities and needs. The study coordinator also ensured that there were regular and productive communications to the team to ensure its’ progress with tasks. Team members must have clearly defined strategies for co-production to structure and attain agreed-upon outcomes [21].

Our team used a framework to guide the conduct of our work together [37], and is one example of how research teams can organize themselves to operationalize concepts important for co-producing research across the research lifecycle [6]. Teams of knowledge users and researchers are striving to explain and evaluate co-production, with a growing theoretical and practical knowledge base in support of a relational approach to research [75–77].

We describe a team approach to collaboration (for example, define, agree upon team governance, research activities and roles) and communication (for example, sustained, consistent) among individuals and groups, and in a range of forms (for example, written, email, spoken). Furthermore, we sought to create opportunities for team members to determine their engagement with tasks as the systematic review proceeded. We assured all team members that they could adjust their preferred levels of engagement over time, and supported people to engage in their preferred manner. Work has been done to understand what knowledge, skills, and attitudes (“competencies”) are helpful for research teams that include families/caregivers as equal partners with researchers, healthcare providers, and decision-makers. While trends in competencies are identified across the different categories of team members, common findings across the team members are attitudes demonstrating the inclination to conduct the work [78].

The strategies we applied to support meaningful engagement of all team members are described elsewhere as creating “space to talk” (team members share views and recognize one another as bringing important knowledge) and “space to change” (in response to the shared knowledge, taking action in the study and how the team works together) [79]. We worked together to meet the needs of our international and interdisciplinary team members, to design an approach to co-production that established a culture and expectation of mutual respect [80].

We note some limitations and strengths of our work. We conducted a self-study as a study within a review and there is no established approach to determine the quality of self-study or a study within a review, as there currently there are no guidelines specific to self-study [81] or studies within a review. In response, we provide clear and detailed descriptions of how we collected and reported data, used available reporting guidelines, and more than one source of data to triangulate and represent the findings of the self-study. Another potential limitation is that we adapted an instrument that was developed for use with researcher-health policy maker partnerships rather than an interdisciplinary team. Our ability to measure co-production using this adapted instrument was limited due to the lack of a validated tool which is a recognized paucity within the co-production literature [82, 83].

We did draw on previous work conducted with international and interdisciplinary teams, and with the leadership of team members who have experience in the conduct of collaborative research. As the study relied on self-reports from participants, our study may be prone to self-reporting bias that include social desirability and recall bias. We were fortunate to have a study coordinator to support participants and ensure that they could participate anonymously. In addition, we may not have fully captured the experiences of team members and a fulsome description of an international and interdisciplinary team experience may not have been conveyed. Our team and circumstances are very specific to the conduct of a systematic review; for this reason, the transferability of findings to other teams and settings may be limited. We were fortunate to have had two Cochrane consumers participate on the team, willing to bring their knowledge and perspectives to the conduct of our work.

There were some lessons learned regarding set up of the initial survey. We did not engage team members in all eleven steps as originally planned. Step 1 “Conduct the search in databases and remove duplicates” was executed according to the study protocol by the librarian. In the future, we might omit this question from the initial survey or revise it to read “Review the search strategy to be used in electronic databases.” Step 2 “Pull full text articles” was done by the study coordinator given it is a mundane task, requires access to an institutional library for access to subscription articles not otherwise publicly available, and we felt that team member expertise and resources could be better used for other tasks. In the future, we would likely omit this item from the initial survey. Step 8 “Conduct analysis” involved adding extracted outcome data to RevMan Web and conducting meta-analyses according to the protocol. We were advised by Cochrane to only allow a limited number of people to revise the report in RevMan Web; therefore, this step was done by the study coordinator and data verified by another team member. In the future, we might omit this question from the initial survey or revise it to read “Discuss the analysis.” Step 9 “Draft the systematic review article” was led by two team members only (JJ, DS) given that there are challenges with involving many people in writing the first draft of an article. In the future, we would discuss how we planned to involve team members in drafting the systematic review prior to deciding whether to include or exclude this item in the initial survey.

## Conclusions

Our mixed methods case study shows that it is feasible to co-produce a systematic review. We used a framework to guide knowledge users and researchers partnering on the systematic review and the conduct of a self-study as a study within a review of a co-production process. We describe the co-production approach used to conduct a systematic review, and report on the experiences and perceptions of international and interdisciplinary team members that include Cochrane consumers. The participants in our self-study reported overall high levels of satisfaction with their engagement in co-production of the systematic review.

We confirm that a relational approach to research is important for co-production. We propose that clearly defined strategies are essential for the co-producing a systematic review such as determining common interests, reaching agreement on the systematic review parameters (study purpose and plan, team organization), and planning and providing supports for communication, collaboration, and opportunities to negotiate participation during the systematic review lifecycle. Although approaches to co-production are assumed to have an influential role in the development of applicable research evidence, high-quality evaluations of co-production processes and outcomes are limited [77, 83, 84]. Our study can contribute to a knowledge base that is a foundation for future methodological studies that investigate co-production within evidence syntheses [28]. An important contribution of our study is that it describes how to structure, report on, and evaluate research processes used in co-producing a systematic review. Further studies that are focused on rigorous evaluations, such as intervention studies, to examine approaches to co-production of systematic reviews by international and interdisciplinary team members and consider team member interests, preferences, and circumstances are needed.

### Supplementary Information

Supplementary Material 1.

Supplementary Material 2.

Supplementary Material 3.

Supplementary Material 4.

Supplementary Material 5.

## Data Availability

The datasets used and/or analyzed during the self-study are available from the corresponding author on reasonable request.
