# Supplementary material for: Co-production of a systematic review on decision coaching: a mixed methods case study within a review
Source: Syst Rev. 2024 Jun 3;13:149. doi: 10.1186/s13643-024-02563-8 (PMC11149211; doi:10.1186/s13643-024-02563-8)
Supplement: Supplementary file 1 — Supplementary Material 1. [file 13643_2024_2563_MOESM1_ESM.docx]

Supplementary File

GRIPP2 long form

| **Section and topic** | | | **Item** | **Reported on page No** |
| --- | --- | --- | --- | --- |
| Section 1: Abstract of paper | | | |  |
| 1a: Aim | | | Report the aim of the study | Yes, see Background |
| 1b: Methods | | | Describe the methods used by which patients and the public were involved | Yes |
| 1c: Results | | | Report the impacts and outcomes of PPI in the study | Yes |
| 1d:Conclusions | | | Summarise the main conclusions of the study | Yes |
| 1e: Keywords | | | Include PPI, “patient and public involvement,” or alternative terms as keywords | Yes, defined and used “knowledge users” |
| Section 2: Background to paper | | | |  |
| 2a: Definition | | | Report the definition of PPI used in the study and how it links to comparable studies | Yes, define knowledge users, co-production, and engagement, and in relation to research studies. |
| 2b: Theoretical underpinnings | | | Report the theoretical rationale and any theoretical influences relating to PPI in the study | Yes, see description of co-production and integrated knowledge translation approach. See “co-production and knowledge synthesis” section. |
| 2c: Concepts and theory development | | | Report any conceptual models or influences used in the study | Yes, as noted above. |
| Section 3: Aims of paper | | | |  |
| 3: Aim | | | Report the aim of the study | See “The research context” with details on team, and our study objectives. |
| Section 4: Methods of paper | | | |  |
| 4a: Design | | | Provide a clear description of methods by which patients and the public were involved | See Methods: Design, Setting and Participants, Procedures for self-study, participant recruitment, engagement and data collection. |
| 4b: People involved | | | Provide a description of patients, carers, and the public involved with the PPI activity in the study | See above. |
| 4c: Stages of involvement | | | Report on how PPI is used at different stages of the study | See above. |
| 4d: Level or nature of involvement | | | Report the level or nature of PPI used at various stages of the study | See above. |
| Section 5: Capture or measurement of PPI impact | | | |  |
| 5a: Qualitative evidence of impact | | If applicable, report the methods used to qualitatively explore the impact of PPI in the study | | See above and Methods sections: Survey instrument, Study documents. |
| 5b: Quantitative evidence of impact | | If applicable, report the methods used to quantitatively measure or assess the impact of PPI | | See above, plus Methods section: Analysis. |
| 5c: Robustness of measure | | If applicable, report the rigour of the method used to capture or measure the impact of PPI | | See above. |
| Section 6: Economic assessment | | | |  |
| 6: Economic assessment | | If applicable, report the method used for an economic assessment of PPI | | n/a |
| Section 7: Study results | | | |  |
| 7a: Outcomes of PPI | | Report the results of PPI in the study, including both positive and negative outcomes | | See Results, reported for each of the objectives of the study. |
| 7b: Impacts of PPI | | Report the positive and negative impacts that PPI has had on the research, the individuals involved (including patients and researchers), and wider impacts | | See above. |
| 7c: Context of PPI | | Report the influence of any contextual factors that enabled or hindered the process or impact of PPI | | See Results, sections: The context for co-production, and results that follow for each of the objectives. |
| 7d: Process of PPI | | Report the influence of any process factors, that enabled or hindered the impact of PPI | | See Results, Objective 3, perceptions of team members about the co-production approach. |
| 7ei: Theory development | | Report any conceptual or theoretical development in PPI that have emerged | | See Discussion for contributions of the study to wider literature. |
| 7eii: Theory development | | Report evaluation of theoretical models, if any | | See Discussion for contributions of the study to wider literature. |
| 7f: Measurement | | If applicable, report all aspects of instrument development and testing (eg, validity, reliability, feasibility, acceptability, responsiveness, interpretability, appropriateness, precision) | | Not applicable. |
| 7g: Economic assessment | | Report any information on the costs or benefit of PPI | | n/a |
| Section 8: Discussion and conclusions | | | |  |
| 8a: Outcomes | Comment on how PPI influenced the study overall. Describe positive and negative effects | | | See Results, Discussion. |
| 8b: Impacts | Comment on the different impacts of PPI identified in this study and how they contribute to new knowledge | | | See Results, Discussion. |
| 8c: Definition | Comment on the definition of PPI used (reported in the Background section) and whether or not you would suggest any changes | | | See Discussion for contributions of the study to wider literature. |
| 8d: Theoretical underpinnings | Comment on any way your study adds to the theoretical development of PPI | | | See Discussion for contributions of the study to wider literature. |
| 8e: Context | Comment on how context factors influenced PPI in the study | | | See Results, Discussion. |
| 8f: Process | Comment on how process factors influenced PPI in the study | | | See Results, Discussion. |
| 8g: Measurement and capture of PPI impact | If applicable, comment on how well PPI impact was evaluated or measured in the study | | | See Discussion and specifically limitations, for contributions of the study to wider literature. |
| 8h: Economic assessment | If applicable, discuss any aspects of the economic cost or benefit of PPI, particularly any suggestions for future economic modelling. | | | n/a |
| 8i: Reflections/critical perspective | Comment critically on the study, reflecting on the things that went well and those that did not, so that others can learn from this study | | | See Discussion and specifically limitations, for contributions of the study to wider literature. |

PPI=patient and public involvement

The development of the original Guidance for Reporting Involvement of Patients and the Public (GRIPP), tackled inconsistent reporting by helping researchers, patients, carers, and the public to improve the quality, consistency, and transparency of patient and public involvement (PPI) reporting, to strengthen the quality of the international PPI evidence base. INVOLVE defines public involvement in research as being carried out with or by members of the public rather than to, about, or for them.

Staniszewska S, Brett J, Simera I, Seers K, Mockford C, Goodlad S et al. GRIPP2 reporting checklists: tools to improve reporting of patient and public involvement in research BMJ 2017; 358 :j3453 doi:10.1136/bmj.j3453

Original GRIPP: Staniszewska S, Brett J, Mockford C, Barber R. The GRIPP checklist: strengthening the quality of patient and public involvement reporting in research. Int J Technol Assess Health Care *2011*;*27*:*391*-*9*. [*doi:10.1017/S0266462311000481*](http://dx.doi.org/10.1017/S0266462311000481) [*pmid:22004782*](http://www.ncbi.nlm.nih.gov/pubmed/?term=22004782).
