# Supplementary material for: Co-production of a systematic review on decision coaching: a mixed methods case study within a review
Source: Syst Rev. 2024 Jun 3;13:149. doi: 10.1186/s13643-024-02563-8 (PMC11149211; doi:10.1186/s13643-024-02563-8)
Supplement: Supplementary file 2 — Supplementary Material 2. [file 13643_2024_2563_MOESM2_ESM.docx]

| **Topic** | **Item** | **Report** |
| --- | --- | --- |
| Title | Concise description of the nature and topic of the study identifying the study as qualitative or indicating the approach (e.g. ethnography, grounded theory) or data collection methods (e.g. interview, focus group) is recommended | Yes |
| Abstract | Summary of key elements of the study using the abstract format of the intended publication; typically includes background, purpose, methods, results and conclusions | Yes |
| **Introduction** |  |  |
| Problem formulation | Description and significance of the problem/phenomenon studied; review of relevant theory and empirical work; problem statement | See Introduction, and sub-sections *Co-production and knowledge synthesis* and  *The research context.* |
| Purpose or research question | Purpose of the study, and specific objectives or research questions | See *The research context.* |
| **Methods** |  |  |
| Qualitative approach and research paradigm | Qualitative approach (e.g. ethnography, case study) and guiding theory if appropriate; identifying the research paradigm (i.e. positivist, constructivist etc) is also recommended; rationale^b^ | See Methods, *Design* and depiction of pragmatic intent with the study. |
| Researcher characteristics and reflexivity | Researchers’ characteristics may influence the research, including personal attributes, qualifications/experience, relationship with participants, assumptions, and/or presuppositions; potential or actual interaction between researchers’ characteristics and the research questions, approach, methods, results and/or transferability | See Methods, *Setting, participants.* |
| Context | Setting/site and salient contextual factors; rationale^b^ | See Methods, *Setting, participants* and *Procedures for self-study participant recruitment, engagement, and data collection.* |
| Sampling strategy | How and why research participants, documents, or events were selected; criteria for deciding when no further sampling was necessary (e.g. sampling saturation); rationale^b^ | See Methods, *Setting, participants* and *Procedures for self-study participant recruitment, engagement, and data collection* and *Survey instrument.* |
| Ethical issues pertaining to humans | Documentation of approval by an appropriate ethics review board, and participant consent, or explanation for lack thereof; other confidentiality and data issues | See Methods, *Setting, participants* for information on ethics review, and *Procedures for self-study participant recruitment, engagement, and data collection.* |
| Data collection methods | Types of data collected; details of data collection procedures including (as appropriate) start and stop of collection and analysis, iterative process, triangulation of sources/methods, modification of procedures in response to evolving study findings; rationale^b^ | See Methods, *Survey instrument* and *Study documents*. We describe adjustments to procedures in Results, *The context for Co-production.* |
| Data collection and instruments | Description of instruments (e.g. survey) and devices used for data collection; if how instruments changed over the course of the study | See Methods, *Survey instrument* and *Study documents*. |
| Units of study | Number and relevant characteristics of participants, documents, or events included in the study; level of participation (could be reported in results) | See Methods, *Setting, participants* for information on ethics review, and *Procedures for self-study participant recruitment, engagement, and data collection.* |
| Data processing | Methods for processing data prior to and during analysis, including transcription, data entry, data management and security, verification of data integrity, data coding, and anonymization and deidentification of excerpts | See Methods, *Analysis*. |
| Data analysis | Process by which inferences, themes etc were identified and developed including the researchers involved in the data analysis; usually references a specific paradigm or approach; rationale^b^ | See Methods, *Analysis*. |
| Techniques to enhance trustworthiness | Techniques to enhance trustworthiness and credibility of data analysis (e.g. member checking); rationale^b^ | See Methods, *Analysis*. |
| **Results/findings** |  |  |
| Synthesis and interpretation | Main findings (e.g. interpretations, themes); might include development of theory or model, or integration with prior research or theory | We report main findings in relation to the objectives of the review and in relation to previous concepts and theory. |
| Links to empirical data | Evidence (e.g. quotes, text excerpts) to substantiate analytic findings | Yes, see in tables and in text, closed and open-ended responses are reported in relation to one another, to substantiate findings. See also rationale for the approach to data reporting in Methods, *Analysis*. |
| **Discussion** |  |  |
| Integration with prior work, implications, transferability, and contributions to the field | Short summary of main findings, explanation of how findings and conclusions connect to support, elaborate on or challenge conclusions of earlier scholarship; discussion of scope of application; identification of unique contributions to scholarship in a discipline or field | Yes, see Discussion summary and discussion points integrate and build on previous work. |
| Limitations | Trustworthiness and limitations of the findings | Yes, see Discussion section, last paragraph. |
| **Other** |  |  |
| Conflicts of interest | Potential sources of influence or perceived influence on study conduct and conclusion; how these were managed | Our team did not identify any conflicts of interest; we engaged a study coordinator to ensure confidentiality and oversight of the study processes. |
| Funding | Sources of funding and other support; role of funders in data collection, interpretation and reporting | Yes, reported (funders did not have a role in the conduct or reporting of the study). |

^a^ The authors of the SRQR created it by searching the literature to identify guidelines, reporting standards, and critical appraisal tools for qualitative research; reviewing the references lists of retrieved sources; contacting experts to gain feedback. The SQRQ aims to improve the transparency of all aspects of qualitative research by providing clear standards for reporting qualitative research.

^b^ The rationale should briefly discuss the justification for choosing that theory, approach, method, or technique rather than other options available, the assumptions and limitations implicit in those choices, and how those choices influence study conclusions and transferability. As appropriate, the rationale for several items might be discussed together.

O'Brien B HI, Beckman TJ, Reed DA, Cook DA. Standards for Reporting Qualitative Research (SRQR). Academic Medicine. 2014;89(9):1245-51
