# Supplementary material for: Co-production of a systematic review on decision coaching: a mixed methods case study within a review
Source: Syst Rev. 2024 Jun 3;13:149. doi: 10.1186/s13643-024-02563-8 (PMC11149211; doi:10.1186/s13643-024-02563-8)
Supplement: Supplementary file 4 — Supplementary Material 4. [file 13643_2024_2563_MOESM4_ESM.pdf]

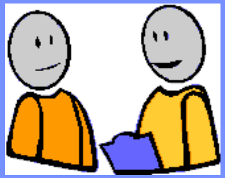

Decision Coaching Systematic Review

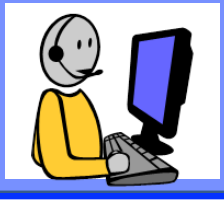

Governance

Executive Committee:
JJ, DS, MS, SK, MC

Steering Committee:
LB, SD, IDG, KL, FL, JK, SK, AL, CR, AR, JF, AD

iKT team:
JJ, DS, IDG, MC

Advisory Committee:
AC, TR, BH, WO

Steering Committee: Terms of Reference

**Role of Steering committee membership**	• The core working group of the research team<br>• To participate in a collaborative process<br>• To provide content-related support, and bring knowledge, skills and experience to the working group throughout the multiple stages of the research process (e.g., steps of systematic review process)
**Method and frequency of communication**	• The research coordinator will provide project background documents<br>• Agendas will be provided in advance of meetings, with identification of key decisions to be made by the steering committee<br>• There will be ongoing opportunities for communication, in a manner that facilitates the function of members in their roles
**Description of workload**	• See Proposal Summary (Appendix A)<br>• Meeting participation<br>• Provision of feedback on key issues to be made in meetings or by email correspondence<br>• There will be opportunities for interested members to meet authorship criteria (see below "Authorship")

**Timelines**	• The Steering committee involvement is anticipated to begin in Oct. 1, 2019 and to end Sept. 31, 2020<br>• Study executive meetings will occur every two weeks in person or by email<br>• Full team updates will occur every month<br>• Full team meetings will occur quarterly (or as needed)
**How advice will be managed**	• Steering committee input will be sought and considered along with that of the Study executive<br>• Disagreements on views will be respectfully and collaboratively managed by a member of the Study executive (JJ)
**Authorship**	• ICMJE (2019) criteria will be used as guidelines for publication authorship:<br>1) Authors must make a substantial contribution to the conception, design, or the acquisition, analysis, or interpretation of data; AND<br>2) Drafting the work or revising it critically for important intellectual content; AND<br>3) Final approval of the version to be published; AND<br>4) Agreement to be accountable for all aspects of the work in ensuring that questions related to the accuracy or integrity of any part of the work are appropriately investigated.<br>• Those that have made other contributions to the work or who meet some of the above criteria will be credited in the acknowledgements but not receive authorship.

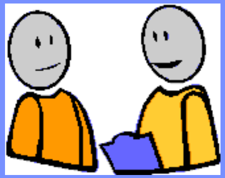

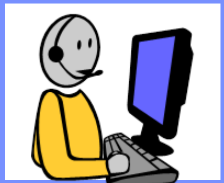

Advisory Committee: Terms of Reference

**Role: Advisory committee members**	• To provide advice for specific decisions during operationalizing the systematic review<br>• Receive monthly updates from the study progress<br>• Be notified of key decisions to be made by the steering committee and invited to provide their advice<br>• Will not be involved in the iKT study
**Timelines**	• The research will be conducted from October 2019 and to Sept 2020
**How advice will be managed**	• Advisory committee input will be sought and considered by the Steering Committee
**Authorship**	• ICMJE (2019) criteria will be used for publication authorship:<br>1)Authors must make a substantial contribution to the conception, design, or the acquisition, analysis, or interpretation of data;<br>2)Drafting/critically revising the work for important intellectual content;<br>3)Final approval of the version to be published;<br>4)Agreement to be accountable for all aspects of the work in ensuring that questions related to the accuracy or integrity of any part of the work are appropriately investigated.<br>• Other contributions will be credited in the acknowledgements
