# Supplementary material for: Co-production of a systematic review on decision coaching: a mixed methods case study within a review
Source: Syst Rev. 2024 Jun 3;13:149. doi: 10.1186/s13643-024-02563-8 (PMC11149211; doi:10.1186/s13643-024-02563-8)
Supplement: Supplementary file 5 — Supplementary Material 5. [file 13643_2024_2563_MOESM5_ESM.docx]

| ***Framework Constructs*** | ***Categories*** | | | ***Key / Icon*** | |
| --- | --- | --- | --- | --- | --- |
| **Who is involved?** | Patients, carers and / or their families | | | 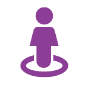 | |
|  | Members of our systematic review team (SR Team) identify as patient partners (n=2), researchers (n=7), trainees (n=4), clinicians (n=2), and other (health systems administrators, educators, member of health advocacy organization) (n=3). | | | 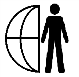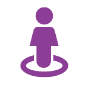 | |
|  | Other stakeholders only | | | 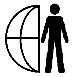 | |
| Fixed 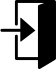 | | | | | |
| **How are people recruited?** | **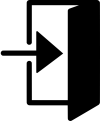**Open | | Fixed | Flexible 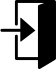 | |
|  |  |  | Flexible | 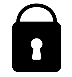 Invite | |
|  | **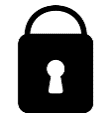**Closed | | Invitation | 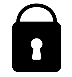 Group | |
|  |  |  | Existing group | 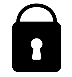 Sample | |
|  |  |  | Purposive sampling |  | |
|  | Other / Unclear | | | **?** | |
|  | | | | | |
| **What happened?**  *Approach?* | One-time | | | 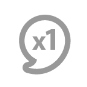 | |
|  | Continuous  Our SR Team consists of a study executive (four members, one includes a patient partner) and an international steering committee (n=13). Our SR Team negotiated our governance structure and agreed upon definitions, the scope of the work, and our arrangements (for example, a terms of reference document). They have been engaged in the entire systematic review process so that the outputs of the research are more likely to be applied in practice and policy. Prior to initiating the conduct of the review, the research coordinator administered an anonymous survey to SR Team members who agreed to participate in the survey so that they could indicate their preferred level of involvement at each step of the review to the research coordinator.  We had ongoing meetings for the entire SR Team. For the study executive, there were more frequent meetings. The patient partner was a member of the four-person executive team that met bi-weekly throughout the entire review.  There were regular and less frequent meetings with the entire SR team (executive and international steering committee). Full team meetings were held approximately every three to four months to facilitate discussion and to ensure all SR Team members had opportunities to provide feedback on all aspects of the review.  There were regular (monthly) email communications to the SR Team about the conduct of the study, and to invite feedback | | | 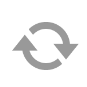 | |
|  | Combined (i.e. both one-time and continuous) | | | 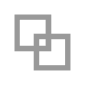 | |
|  | | | | | |
| **What happened?**  *Methods?* | Direct interaction  There were some smaller, in-person meetings among some members of the SR Team in the spring and summer of 2019. In-person meetings were planned but had to be held virtually due to the pandemic. There were four virtual SR Team meetings (every 3 months, over a 1.5 year time period). | | | **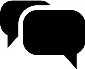** | |
|  | No direct interaction | | | **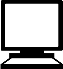** | |
|  | | | | | |
| **Stage & Level?**  **SEE BELOW FOR EXPLANATIONS** 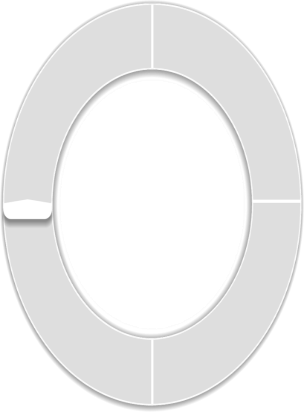 1  Develop question  2  Plan methods  3  Write & publish protocol  4  Develop search  5  Run search  6  Select studies  7  Collect data  8  Assess risk of bias  9  Analyze data  10  Interpret findings  11  Write & publish review  *12*  *Knowledge translation & impact*  **ACTIVE stages of a systematic review** |  | Leading  All team members, including the patient partners participated in key decisions relating to the methods and execution of the review .  There were specialized team members involved in the search for articles (librarian scientist); and a subset of researchers who developed the data extraction form and collected data, summarize participant and study characteristics, and analysis, all on items agreed to by team members, that include the patient partners. One patient partner who was on the study executive excused herself from engaging in the risk of bias assessment of included articles. Otherwise, this patient partner participated in all steps of the review, attended all meetings where every step of the review was discussed, and provided feedback. The patient partners chose the extent of their engagement at every stage of the systematic review based on their interests and availability.  Prior to initiating the conduct of the review, an anonymous survey was administered to SR Team members so that they could indicate their preferred level of involvement at each step of the review to the research coordinator (leading, controlling, influencing, contributing).  Various SR Team members contributed at each stage of the research in different ways, and according to their preferences, with all team members at least participating in “contributing”. | | | Lead |
|  |  | Controlling  At different stages of the review, SR Team members moved to a controlling role, meaning that they preferred to have a varied role and to advise or be informed | | | Control |
|  |  | Influencing | | | Influen |
|  |  | Contributing | | | Contrib |
|  |  | Receiving | | | Receive |
|  | Top & tail approach? No | | | 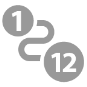 | |

**FIGURE 1: The ACTIVE framework of involvement in a systematic review**
